# Dysfunction of Autophagy: A Possible Mechanism Involved in the Pathogenesis of Vitiligo by Breaking the Redox Balance of Melanocytes

**DOI:** 10.1155/2016/3401570

**Published:** 2016-11-29

**Authors:** Zhuhui Qiao, Xiuxiu Wang, Leihong Xiang, Chengfeng Zhang

**Affiliations:** Department of Dermatology, Huashan Hospital, Fudan University, Shanghai, China

## Abstract

Vitiligo is a common chronic acquired pigmentation disorder characterized by loss of functional melanocytes from the epidermis and follicular reservoir. Among multiple hypotheses which have been proposed in the pathogenesis of vitiligo, autoimmunity and oxidative stress-mediated toxicity in melanocytes remain most widely accepted. Macroautophagy is a lysosome-dependent degradation pathway which widely exists in eukaryotic cells. Autophagy participates in the oxidative stress response in many cells, which plays a protective role in preventing damage caused by oxidative stress. Recent studies have enrolled autophagy as an important regulator in limiting damage caused by UV light and lipid oxidation, keeping oxidative stress in a steady state in epidermal keratinocytes and maintaining normal proliferation and aging of melanocytes. Impairment of autophagy might disrupt the antioxidant defense system which renders melanocytes to oxidative insults. These findings provide supportive evidence to explore new ideas of the pathogenesis of vitiligo and other pigmentation disorders.

## 1. Introduction

Vitiligo is an acquired pigmentary disorder of the skin and mucous membranes that is characterized by circumscribed, depigmented macules and patches. The worldwide prevalence of vitiligo ranges between 0.5 and 2% [1]. The highest incidence of the condition has been recorded in India, followed by Mexico and Japan [2]. Although vitiligo does not affect individual survival of patients, the change of the appearance may cause physical handicaps which seriously interfere with the lives of patients [3, 4]. In the past decades, a number of mechanisms such as oxidative stress, autoimmune, autocytotoxicity [5, 6], melanocytorrhagy [7], neural, and genetic factors [7] have been proposed for the pathogenesis of vitiligo. Oxidative stress hypothesis indicated imbalanced redox state of the vitiliginous skin. This results in the dramatic production of reactive oxygen species (ROS), such as hydrogen peroxide (H_2_O_2_). ROS oxidize components of cells, leading to melanocyte destruction and creating depigmented macules [8]. Autoimmune theory showed that melanocytes are killed by autoimmune effector mechanisms, either memory cytotoxic T cells or autoantibodies directed to melanocyte surface antigens, as a result of tolerance breakage [9]. It is widely known that vitiligo can be associated with several autoimmune diseases, including autoimmune thyroid diseases [10], alopecia areata [11], halo nevi [12], and Addison's disease [13]. Furthermore, it has been shown that autoimmune thyroid diseases, especially Hashimoto thyroiditis, are the most common vitiligo-associated disorders [14]. The melanocythorragic hypothesis was proposed by Gauthier et al. in 2003 and they mentioned that nonsegmental vitiligo (NSV) occurs due to “melanocytorrhagy” or a chronic melanocyte detachment and loss caused by trauma and other stressors including catecholamines, ROS, or autoimmune elements [15]. This theory combined the concepts from other theories mentioned before to elaborate a single integrated explanation of vitiligo pathogenesis. The neural hypothesis suggested that accumulation of a neurochemical substance decreases melanin production. Abundant norepinephrine (NE) as an extrinsic factor secreted by nerve endings or keratinocytes leads to direct impairment of melanocytes differentiated from neural crest cells [16]. The link between vitiligo and the activity of monoaminergic systems has been found based on the finding that levels of norepinephrine (NE), epinephrine (E), normetanephrine (NMN), metanephrine (MN), homovanillic acid (HVA), and 5-hydroxyindolacetic acid (5-HIAA) were significantly higher in patients with vitiligo compared to controls [17]. The current dogma is that there is a genetic component that renders the melanocyte fragile and susceptible to apoptosis that in turn predisposes individuals to developing the disease. Precipitating factors including sunburn, pregnancy, stress, and exposure to cytotoxic compounds can induce the fragile melanocytes to initiate programmed cell death or apoptosis when compared with the normal melanocyte [18]. Among the theories mentioned above, oxidative stress plays an extremely important role in the onset and progression of the disease [19]. In this article, we aim to summarize the latest findings concerning the association among oxidative stress, autophagy, and vitiligo.

## 2. Oxidative Stress and Vitiligo

Upon excessive oxidative stress, human bodies may suffer from various harmful factors, including highly active molecules such as active oxygen free radicals and ROS, which leads to impairment of redox balance and results in tissue damage. ROS can attack melanocytes and interfere with normal metabolism, proliferation, and differentiation of melanocytes, causing cell apoptosis and defects [20]. Increased levels of ROS might impair the function of mitochondrial. Excessive accumulation of ROS caused by chemical or physical stimulation in epidermis of vitiligo patients leads to destabilization, synthesis, and circulatory disorders of lipids in melanocytes, resulting in damage of mitochondria electron transport chain, and more ROS production, further forming a vicious circle and destroying melanocytes [21]. Patients with vitiligo accumulate up to 10^−3^ mol/L concentrations of H_2_O_2_ in their epidermis, which in turn affects many metabolic pathways in this compartment, including the synthesis and recycling of the cofactor (6R)-l-erythro-5,6,7,8-tetrahydrobiopterin (6BH4) [22]. In addition, it has been found that mitochondria appeared swollen and lack a normal and organized ultrastructure of internal and external membrane systems in keratinocytes from perilesional skin of vitiligo patients and the number of Langerhans cells increased in varying differentiative phases, in lesional skin of vitiligo patients, indicating that mitochondrial damage is associated with the increase in ROS production and, hence, oxidative stress decreased total antioxidant capacity [23]. Besides, alteration in the mitochondrial distribution exists in melanocytes from vitiligo patients, suggesting that the impaired mitochondrial activity may account for the increased susceptibility to prooxidants [24]. In addition, oxidative stress can increase the production of toxic intermediates during melanin synthesis, which promotes the release of catecholamine (CA) and then triggers the damage of melanocytes [25].

Among the great variety of ROS, H_2_O_2_ has a pivotal role in the onset and progression of vitiligo [5]. Several studies have shown the level of catalase decreased in the skin of vitiligo patients and the concentration of H_2_O_2_ in the epidermis was much higher than normal. Suction blister roofs taken from the involved and uninvolved epidermis of patients with vitiligo showed a consistent reduction in levels of catalase compared to normal healthy controls of matched photo-skin types. A decrease in catalase activity is expected to increase the concentration of hydrogen peroxide in the epidermis of these patients [26]. Elevated H_2_O_2_ levels were demonstrated in vitiligo patients compared with healthy controls by utilizing Fourier-transform Raman spectroscopy, providing in vivo evidence for epidermal H_2_O_2_ accumulation within the entire skin of patients with vitiligo. These results are strongly supported by the in vitro observations using epidermal cell extracts, as well as melanocyte and keratinocyte cell cultures established from lesional and nonlesional epidermis of patients with vitiligo [27].

Keratinocytes transfer H_2_O_2_ to the neighboring melanocytes [28], thereby inactivating various antioxidant enzymes, including catalase (CAT), glutathione S-transferase (GST), acetylcholinesterase (AchE), and methionine sulfoxide reductase A (MsrA). The activity of these enzymes in lesional skin of patients with vitiligo was significantly reduced [29–31], so as to further aggravate the accumulation of ROS. Studies have indicated that the destruction of antioxidant systems in patients with vitiligo made the melanocytes more vulnerable to damage caused by oxidative stress [32]. In contrast, other researches on oxidative stress in vitiligo showed that there was a statistically significant increase in the levels of malondialdehyde (MDA), superoxide dismutase (SOD), glutathione peroxidase (GSH-PX), and other antioxidant enzymes in patients with vitiligo compared with control group [33]. Furthermore, some evidence confirmed that low concentration of H_2_O_2_ could affect the regulation of human pigmentation by increasing melanin synthesis and melanosome transfer, while this activity was downregulated by high concentrations of H_2_O_2_, suggesting that oxidation levels differentially impact melanocytes [34].

Keap1 (Kelch-like ECH-associated protein 1)/Nrf2 (nuclear factor (erythroid-derived 2)-like 2)/ARE (antioxidant responsive element) signaling pathway is the antioxidant response that provides cellular antioxidants and detoxifying enzymes [35]. The key transcription factor Nrf2 regulates the expression of phase II detoxifying and antioxidant genes by binding to the ARE sequence [36]. Under basal conditions, the transcription factor Nrf2 is located in the cytoplasm of the cell bound to Keap1 [37]. When cells are under oxidative stress, the inducers disrupt the cytoplasmic complex between the actin-bound protein Keap1 and the transcription factor Nrf2, thereby releasing Nrf2 to migrate to the nucleus where it activates ARE of phase II genes and accelerates their transcriptions [38]. Recent studies have identified an important role for the Nrf2 pathway in maintaining skin homeostasis in epidermis [39]. Phase II genes encode heme oxygenase-1 (HO-1), catalase (CAT), superoxide dismutase (SOD), glutathione-S-transferase (GST), glutathione peroxidase (GPx), NAD(P)H quinone oxidoreductase 1 (NQO1), glutamate-cysteine ligase catalytic subunit (GCLC), and glutamyl cystine ligase modulatory subunit (GCLM) [40]. Previous study showed that Nrf2-ARE pathway and its downstream antioxidants are crucial for melanocytes to cope with H_2_O_2_-induced oxidative damage [41]. Investigation on the integrity of phase II detoxification pathway in the epidermis of vitiligo patients showed that the transcript levels of Nrf2 as well as the downstream detoxification genes NQO-1, GCLC, and GCLM are upregulated in the lesional epidermis compared with the matched nonlesional skin [42]. Meanwhile, several evidences demonstrated that the induction of HO-1 by activating the Nrf2-ARE pathway protected melanocytes against H_2_O_2_-induced toxicity and that upregulation of Nrf2 expression diminished the H_2_O_2_-induced apoptosis of human melanocytes [43]. Furthermore, genetic studies in Chinese Han population demonstrated that Nrf2 gene polymorphisms are associated with the susceptibility to vitiligo [44, 45]. Taken together, such evidence indicated that the Nrf2-ARE signaling pathway is altered in vitiligo melanocytes and alteration of this pathway might be involved in the pathogenesis of vitiligo. Therefore, impairment of the Nrf2-ARE signaling pathway in vitiligo melanocytes might lead to a dysfunction of redox balance, which provides a reasonable explanation as to why the melanocytes in vitiligo patients are hypersensitive to H_2_O_2_-induced oxidative stress [41].

## 3. Oxidative Stress and Autophagy

Autophagy, also referred to as “self-eating,” is a lysosome-dependent degradation pathway that widely exists in eukaryotic cells. In severe conditions such as hunger, infection, or stress, it can regulate the degradation of long-life proteins and organelles in cells and then the degradation products are recycled [46]. In addition, autophagy also plays an important role in cell growth, cell immunity, tissue remodeling, and adaptability to the environment [47]. There are roughly three classes of autophagy: macroautophagy, microautophagy, and chaperone-mediated autophagy. Macroautophagy is thought to be the major type of autophagy and it has been studied most extensively compared to microautophagy and chaperone-mediated autophagy [46]. Autophagy can be upregulated in response to extra or intracellular stress and signals such as starvation, growth factor deprivation, endoplasmic reticulum (ER) stress, accumulation of unfolded proteins, and infection [48]. One of the best characterized substrates of selective autophagy is p62, which is also known as sequestosome 1/SQSTM1. P62 is a ubiquitously expressed cellular protein, which is conserved in animals but not in plants and fungi [46]. Impairment of autophagy is accompanied by accumulation of p62. This leads to the formation of large aggregates, which include p62 and ubiquitin [49]. Functions of p62 may determine whether cells survive by activating the TNF receptor-associated factor 6- (TRAF6-) NF-kB pathway or die by facilitating the aggregation of caspase-8 and downstream effector caspases [50]. On the other hand, p62 interacts with the Nrf2-binding site on Keap1, a component of Cullin3-type ubiquitin ligase for Nrf2. This interaction stabilizes Nrf2 and activates the transcription of Nrf2 target genes, including a battery of antioxidant proteins [51, 52]. It is thus possible that excess accumulation or aggregation of p62 leads to hyperactivation of these signaling pathways [46].

Autophagy has been proved to participate in the removal of toxic molecules produced after oxidative stress. Oxidatively modified proteins are usually considered degraded more or less exclusively by the proteasome system. Rather, evidence suggests that moderately or heavily oxidized proteins are endocytosed and enter the endosomal/lysosomal system, indicating that the proteins modified by oxidizing stressors are degraded by the proteasome and autophagosomal/lysosomal pathways [53]. More and more evidence has been raised suggesting the balance between autophagy and oxidative stress is essential to maintain the function of cells, as well. He et al. found that short exposure to oxidative stress could induce autophagy in myocardial cells, while long-term oxidative stress stimulated the inhibition of autophagy, which leaded to the death of cardiac muscle cells. Therefore, stability of autophagy plays an important role in protecting different types of cells from oxidative stress [54]. Recent study demonstrated that the pathological changes observed in autophagy-defective livers were due, at least in part, to persistent activation of Nrf2 by the excess accumulation of p62, resulting in the appearance of destructive phenotypes and liver injury, proposing that, in such pathological conditions, the high levels of p62 associated with the suppression of autophagy might result in activation of Nrf2 [51].

Recently, autophagy in skin cells has been studied. External and internal sources of oxidative stress include UVR/IR, pollution (environment), lifestyle (exercise and diet), alcohol, and smoking all of which may potentially have impacts on skin [55]. All these factors, leading to an increase in ROS generation and/or a reduction in the antioxidant capacity, contribute to oxidative stress, which exposes the skin cells to the formation and accumulation of irreversibly damaged proteins, lipids, nucleic acids, and carbohydrates [56]. Human skin is exposed to environmental insults such as UV light that cause oxidative damage to macromolecules. In response to oxidative stress, cells activate the Nrf2 antioxidant response that provides cellular antioxidants and detoxifying enzymes [35]. Meanwhile, epidermal keratinocytes activate autophagy in response to UVA and UV-oxidized phospholipid [57]. Genetic elimination of autophagy resulted in massive accumulation of protein aggregates in stressed cells, elevation of Nrf2 target gene expression, and strikingly a significant rise in various oxidized species of phospholipids, implying that during homeostasis autophagy prevents accumulation of oxidized phospholipids, as well as overexpression of Nrf2 target genes in keratinocytes [57].

**Figure 1:**
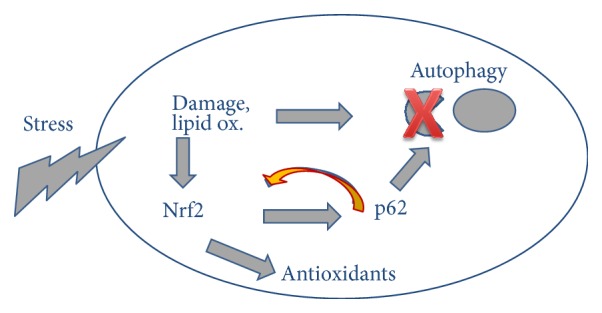
P62, a target gene AND activator of Nrf2.

In autophagy-deficient cells, an accumulation of the adapter protein p62 can be observed frequently [57]. It has been described that lipoxidative stress can lead to protein aggregates that are positive for p62, so called “p62 bodies” in various tissues [51]. P62 is however not only the adapter between cargo and the autophagic machinery but also a component of the Nrf2 pathway. It is not only a target of Nrf2, but its accumulation can compete with Nrf2 for binding to its cytosolic anchor, which then leads to increased Nrf2 nuclear translocation and activity (Figure 1). Excessive accumulation of p62 in autophagy-deficient mice and excessive activation of Nrf2 pathway could cause liver cell damage, which is consistent with the disease such as drug hepatitis and hepatocellular carcinoma [51]. In addition, blockage of autophagy in mice keratinocytes or melanocytes led to substantial accumulation of high molecular weight protein p62, excessive activation of the antioxidant Nrf2 pathway, and high expression of a large number of harmful macromolecular protein and lipid oxidation of phosphoric acid [57, 58]. Thus, normal activation of the Nrf2 pathway can protect cells from oxidative insults, while long-term and excessive activation of the Nrf2 pathway can no longer protect the cells but cause tissue damage.

External and internal sources of oxidative stress expose the cells to the formation and accumulation of irreversibly damaged proteins and lipids, activating the Nrf2 antioxidant response that provides cellular antioxidants and detoxifying enzyme. Impairment of autophagy leads to accumulation of high molecular weight protein p62, which can compete with Nrf2 for binding to its cytosolic anchor, leading to increased Nrf2 nuclear translocation and activity. Excessive activation of the Nrf2 pathway may no longer protect the cells but cause tissue damage.

## 4. The Association among Autophagy, Oxidative Stress, and Vitiligo

Skin is the largest human organ and is directly exposed to environmental oxidant stress, including UVA and oxidized lipids generated by UV irradiation. Melanocytes settle down in the epidermis and act as the guard to photo-protection and thermoregulation by packaging melanin pigment into melanosome and delivering them to neighboring keratinocytes. Epidermal melanocytes are particularly vulnerable to excessive ROS production owing to their specialized function: melanin synthesis, which is stimulated by sun exposure, during the process of tanning, and by inflammation that results in postinflammatory hyperpigmentation [59]. Even though the mechanism of melanocyte loss in vitiligo has not yet been clarified, a rising number of studies provided evidence that dysfunction of autophagy may serve as a vital factor. Recently, more and more research has focused on the possible associations between autophagy and melanosome biogenesis, formation, and destruction [59, 60]. Small interfering RNA-based screens identified autophagy genes as having an impact on melanogenesis and heterozygosity for the autophagy regulator beclin-1 results in altered fur color of mice [61]. In addition, members of the autophagic machinery have been proposed to have a role in the formation and maturation of melanosome [62, 63]. However, direct evidence for this claim is still lacking. Murase et al. explored the involvement of autophagy in determining skin color by regulating melanosome degradation in keratinocytes. Melanosome accumulation in keratinocytes was accelerated by treatment with lysosomal inhibitors or with small interfering RNAs specific for autophagy-related proteins, which are essential for autophagy. Furthermore, consistent with the alterations in skin appearance, the melanin levels in human skin cultured ex vivo and in human skin substitutes in vitro were substantially diminished by activators of autophagy and enhanced by the inhibitors, revealing that autophagy has a pivotal role in skin color determination by regulating melanosome degradation in keratinocytes and thereby contributes to the ethnic diversity of skin color [60]. In addition, Zhang et al. showed that mouse melanocytes lacking the autophagy protein Atg7 undergo premature senescence in vitro and accumulate products of oxidative damage, despite activation of the redox response [58]. Taken together, evidence mentioned above indicated that autophagy has implications for melanocyte dysfunction and manifestations of skin pigmentary disorders.

**Figure 2:**
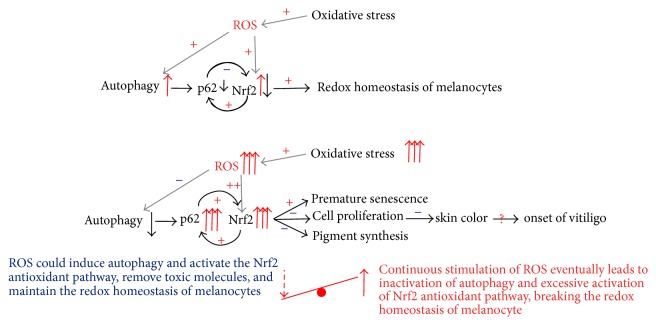
Oxidative stress, autophagy, and vitiligo.

In summary, oxidative stress is currently recognized as one of the most important theories concerning pathogenesis of vitiligo, while autophagy is involved in response to oxidative stress and plays a protective role in the damage induced by oxidative stress in melanocytes. Autophagy is required for suppressing the activation of the Nrf2-dependent stress response, maintaining the proliferative potential and preventing premature senescence of melanocytes. It has been described that controlled p62 levels and p62 phosphorylation are needed for ordered cell cycle progression, so hyperexpression of p62 may well interfere with cell cycle [64]. We speculate the accumulation of p62 and consequently Nrf2 dysregulation might be a sign of impaired redox household and cell cycle machinery and contribute to the observed decrease in the proliferative potential of autophagy-deficient melanocytes. Disturbance of autophagy might release the control of Nrf2 and indirectly triggers an antistress response. If this response is too strong, premature senescence is likely to be induced (Figure 2).

Under oxidative stress, ROS produced in melanocytes could induce autophagy and activate the Nrf2 antioxidant pathway, remove toxic molecules, and maintain the redox homeostasis of melanocytes, while continuous stimulation of ROS might eventually lead to inactivation of autophagy and excessive activation of Nrf2 antioxidant pathway and break the redox homeostasis of melanocytes, resulting in premature senescence, decreased proliferation, and pigment synthesis, which may be involved in the onset of vitiligo.

## 5. Conclusion

Oxidative stress plays an important role in initiating the destruction of melanocytes and impairment of redox balance could be one possible mechanism of vitiligo. Since autophagy possesses a protective and restorative function to the damage induced by oxidative stress, deficiency of autophagy might lead to alteration of cell functions such as proliferation, senescence, and ROS scavenging. On the basis of the similarity between Atg7-deficient and vitiligo phenotypes, specifically with respect to the activation of Nrf2 regulated genes, oxidative stress, and premature senescence, it is very likely that autophagy-deficient melanocytes and vitiligo melanocytes share defective cellular redox regulation, increased membrane lipid oxidation, and premature senescence [65]. However, whether there exists dysfunction of autophagy in vitiligo patients and whether autophagy plays a role in the pathogenesis of vitiligo remain to be established. Additional studies are required to establish an unambiguous role for autophagy in melanocyte biology and skin pigmentation.
